# Structural basis for catalytically restrictive dynamics of a high-energy enzyme state

**DOI:** 10.1038/ncomms8644

**Published:** 2015-07-03

**Authors:** Michael Kovermann, Jörgen Ådén, Christin Grundström, A. Elisabeth Sauer-Eriksson, Uwe H. Sauer, Magnus Wolf-Watz

**Affiliations:** 1 Department of Chemistry, Chemical Biological Centre, Umeå University, Umeå 90187, Sweden

## Abstract

An emerging paradigm in enzymology is that transient high-energy structural states play crucial roles in enzymatic reaction cycles. Generally, these high-energy or ‘invisible' states cannot be studied directly at atomic resolution using existing structural and spectroscopic techniques owing to their low populations or short residence times. Here we report the direct NMR-based detection of the molecular topology and conformational dynamics of a catalytically indispensable high-energy state of an adenylate kinase variant. On the basis of matching energy barriers for conformational dynamics and catalytic turnover, it was found that the enzyme's catalytic activity is governed by its dynamic interconversion between the high-energy state and a ground state structure that was determined by X-ray crystallography. Our results show that it is possible to rationally tune enzymes' conformational dynamics and hence their catalytic power—a key aspect in rational design of enzymes catalysing novel reactions.

## Abstract (web summary)

Adenylate kinase (AdK) plays a key role in cellular energy homeostasis by catalysing the reversible magnesium-dependent formation of ADP from AMP and ATP. Here the authors present a detailed analysis of adenylate kinase's conformational dynamics and characterize a high-energy state of AdK indispensable for catalysis.

## Graphical abstract

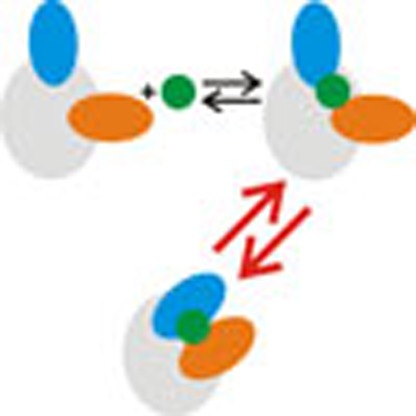

Enzymatic catalysis changes the timescales of otherwise slow chemical reactions, making them commensurate with those of biological processes. An enzyme's capacity to enhance the rate of its catalysed reaction depends on its ability to reduce the free energy of transition state compound(s) (that is, formation of Michaelis' complexes), activate functional groups, dehydrate active sites and align substrates in an optimal geometry for reaction. These functionalities are linked to the enzymes' conformational dynamics, which are defined in terms of the time-dependent displacement of atomic coordinates. In an enzymatic reaction cycle, it is possible to identify stable ground states that can be characterized experimentally using techniques such as X-ray crystallography and nuclear magnetic resonance (NMR) spectroscopy. Such ground states include substrate-free and substrate-bound states, but high-resolution ground state structures alone cannot fully explain enzymes' catalytic power. Enzymes dynamically access structures with higher free energies than those of the stable ground states. These high-energy states are transient and not sufficiently populated to permit direct spectroscopic observation. Consequently, the dynamic interconversions of enzymes' ground and high-energy states have mainly been characterized indirectly via NMR relaxation dispersion experiments^1^,^2^, which have been used to unravel the catalytic cycles of cyclophilin A (CypA)^3^,^4^, adenylate kinase (AdK)^5^, dihydrofolate reductase (DHFR)^6^ and RNase A^7^, and also to explore the structures of sparsely populated folding intermediates^8^. It has been demonstrated that certain enzymes' rates of catalytic turnover are limited by their conformational dynamics^4^,^5^,^6^,^9^. AdK catalysis, which plays a vital role in cellular energy homeostasis by catalysing the reversible magnesium-dependent formation of ADP from AMP and ATP^10^, is dependent on distinct ATP- and AMP-binding subdomains (ATPlid and AMPbd; Fig. 1a). It has been suggested that apo AdK exists in a dynamic equilibrium between open- and closed-like conformational states^11^,^12^, and that certain mutations can modulate this equilibrium^13^. The turnover rate of AdK is limited by product release, and it has been shown that the microscopic step accountable for this slow release is the re-opening of the substrate-binding subdomains^5^,^14^ (Fig. 1a). Thus, the conformational dynamics required for AdK catalysis is given by *k*_conf_, which is defined as the sum of forward and reverse reaction rates (*k*_conf_=*k*_close_+*k*_open_). Here we report the direct NMR-driven observation and molecular characterization of a catalytically indispensable high-energy state of AdK. We also provide evidence for an essential coupling between its conformational dynamics and catalytic turnover. Our results show that it is possible to tune catalytically relevant enzyme dynamics, which is essential for the *de novo* design of enzymes^15^.

## Results

### Selection of mutation sites

**Figure 1:**
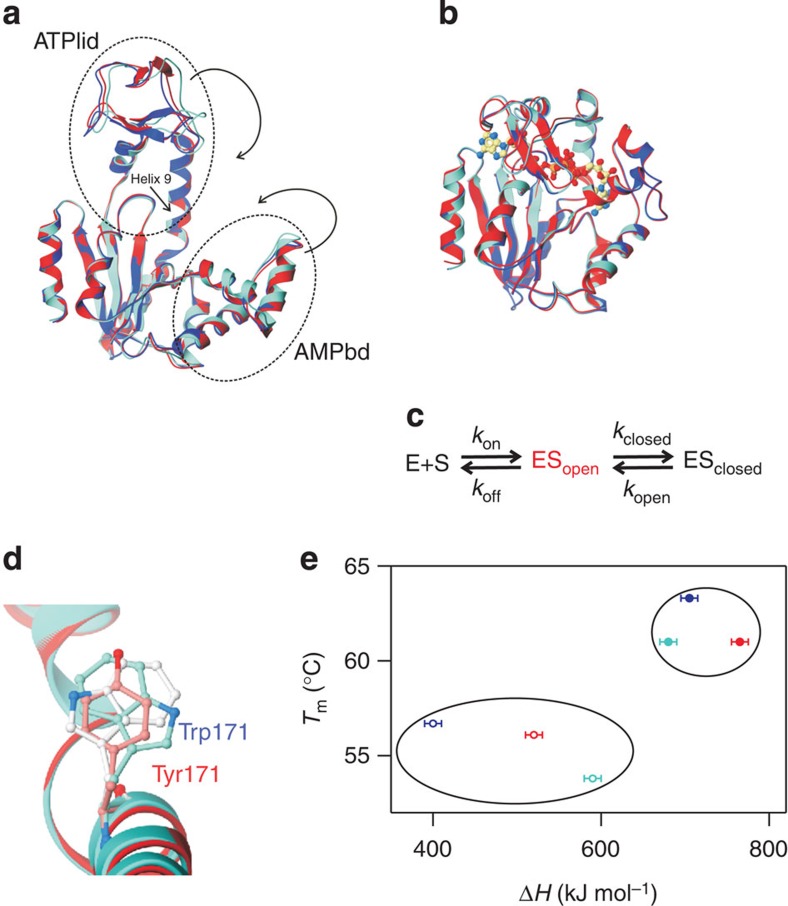
Ground state structures, energetics and kinetic mechanism of AdK variants. (**a**) Ribbon diagrams of the crystal structures of the open apo forms of the wild type (4AKE, red), P177A (4X8H, blue) and Y171W (4X8M, cyan) AdK variants. The ATPlid and AMPbd subdomains are labelled and the trajectories for domain closure into the catalytic closed conformation are indicated with arrows. (**b**) Crystal structures of the closed Ap5a-bound forms of the wild type (1AKE), P177A (4X8L) and Y171W (4X8O) AdK variants (colour code as in **a**). (**c**) A minimal kinetic mechanism of AdK catalysis indicates the presence of a transient high-energy state (highlighted in red) that is simultaneously open and substrate bound. (**d**) The side chain of Trp171 in Y171W samples two different conformations (cyan and white) in the Ap5a-bound state (4X8O). The side-chain orientation of Tyr171 for wild type (light red) is shown as reference. Ribbon colours as in **a**. (**e**) The global enthalpies of unfolding vary considerably for the apo states (open circles) of the wild type, P177A and Y171W, whereas those of the Ap5a-bound states (closed circles) are more closely clustered. Colour code as in **a**.

To directly observe a transient high-energy state in the AdK reaction cycle by NMR spectroscopy, it is essential to slow down the enzyme's conformational dynamics such that the slow exchange limit is approached. Slow exchange on the NMR timescale means that the rate of interconversion (*k*_conf_=*k*_close_+*k*_open_) between states is well below the difference in their chemical shifts. Under such conditions, discrete peaks corresponding to the different states of the exchanging system can be observed. Conversely, in the fast exchange limit, the exchanging system produces only one averaged peak. We have previously identified alpha helix 9 of the ATPlid domain (Fig. 1a) as a key element in the allosteric modulation of the opening and closing dynamics of AdK, and shown that a single amino-acid replacement is sufficient to shift the equilibrium between the open and closed states^13^. We speculated that replacing various residues in helix 9 of AdK might generate enzyme variants whose conformational dynamics are modulated in a way that would enable the direct detection of functional high-energy states by NMR. We selected two single-point mutations of AdK suitable to investigate this hypothesis. The Pro177 to Ala (P177A) mutation was chosen because proline at position 177 is conserved in AdK^11^ and introduces a kink in helix 9 whose importance in the enzyme's opening/closing dynamics has been demonstrated by all-atom molecular dynamics (MD) simulations^16^. The Tyr171 to Trp (Y171W) mutation was selected because it was anticipated that this mutation would modulate the enzyme's dynamics and facilitate stopped-flow measurements of the enzyme's ligand binding kinetics.

### Characterization of ground state structures

AdK populates two main structural ground states: an open substrate-free state and a closed Ap5a-bound state. P1,P5-di(adenosine-5′)pentaphosphate (Ap5a) consists of one ATP and one AMP molecule bridged by a phosphoryl group, and is a good structural mimic of the enzyme's two native substrates. The relevance of this molecule is underscored by the fact that the free energy for its binding to AdK^17^ is equal to the sum of the AdK-binding free energies of AMP and ATP^18^. The ground state structures of the P177A and Y171W variants in their apo- and Ap5a-bound states were determined by X-ray crystallography (Table 1). The apo and holo structures of both variant enzymes are very similar to that of the wild type (Fig. 1a,b) with only minor differences in the conformations of their AMPbd and ATPlid domains. The catalytic cycles (Fig. 1c) of the two AdK mutants are thus supported by the same underlying structural framework as that of the wild-type enzyme. Notably, the electron density of Trp171 shows that the side-chain samples two rotamer conformations in the Ap5a-bound state (Fig. 1d and Supplementary Fig. 1a,b), and the wild-type Y171 conformation is partially overlapping both of these Trp171 rotamers (Fig. 1d). Analysis of the *C*_α_ displacements in the X-ray structures of the apo- (Supplementary Fig. 1c) and Ap5a-bound states compared with wild-type AdK (Supplementary Fig. 1d) of the two AdK variants revealed that the displacements of the apo states were significantly more variable than those of the Ap5a-bound states. That is to say, the apo states are more conformationally heterogeneous. NMR chemical shift perturbation analysis also indicated that the conformations of the apo states (Supplementary Fig. 1e,f) differed more than those of the Ap5a-bound states (Supplementary Fig. 1g–i), showing that the apo states exhibit greater structural heterogeneity both in solution and in the crystals. In keeping with these findings, circular dichroism (CD) spectroscopy indicated that the apo states had a broader distribution of thermodynamic unfolding stabilities (Fig. 1e and Supplementary Fig. 1j) than their Ap5a-bound counterparts (Fig. 1e and Supplementary Fig. 1k).

### Ap5a-bound Y171W populates two interconverting structures

**Figure 2:**
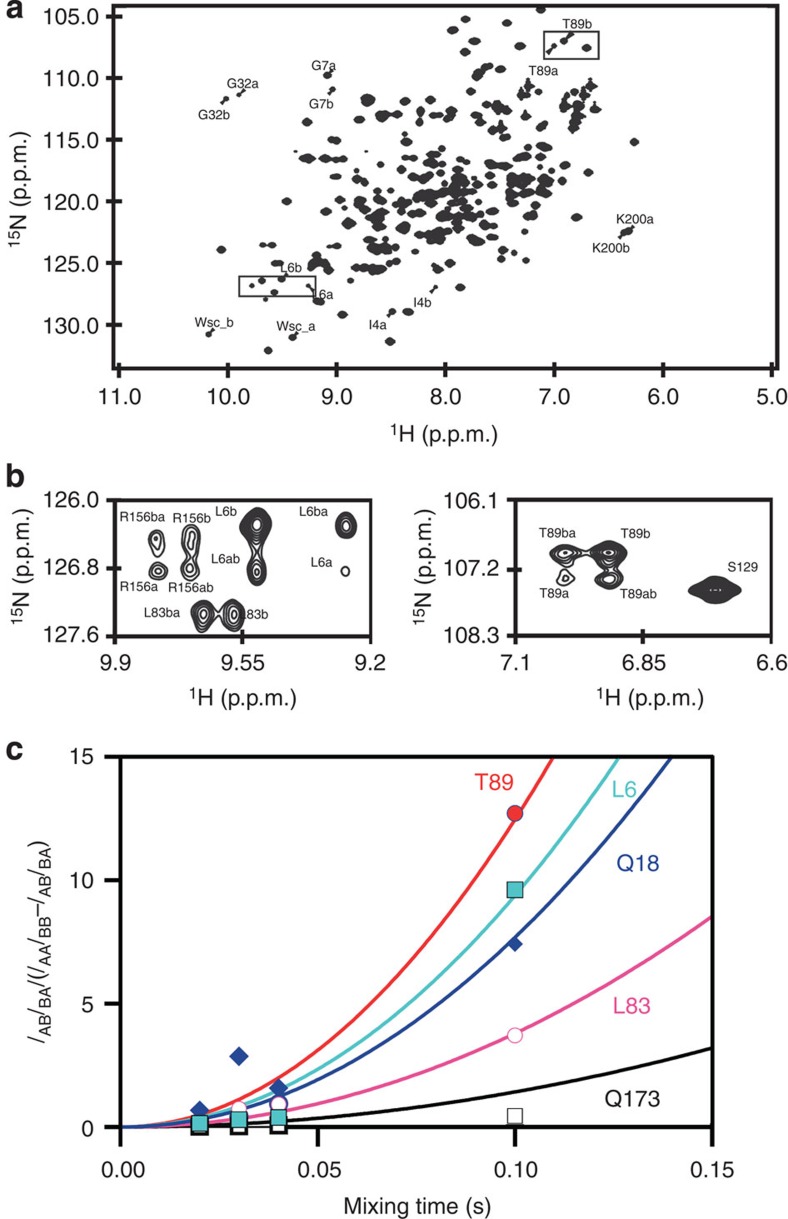
Observation of exchange kinetics between two Ap5a-bound states of Y171W. (**a**) Dual peaks in the ^1^H–^15^N HSQC spectrum of Ap5a-bound Y171W reveal the presence of two structural states. Enlargements of the two spectral regions enclosed by dotted boxes are presented in subfigure **b**. (**b**) Exchange peaks in ZZ-exchange spectra of Ap5a-bound Y171W indicate the interconversion of the two structural states. Diagonal peaks are indicated by ‘a' and ‘b', whereas exchange cross-peaks are labelled as ‘ab' and ‘ba', respectively. (**c**) The rate constant for conformational exchange, *k*_conf_, between the two structural states (*k*_conf_ =25±10 s^−1^) was determined by quantitative analysis of ZZ-exchange data for the displayed residues at 15 °C.

Inspection of the proteins' NMR spectra indicated that the Y171W mutation significantly reduced the rate constant of the enzyme's conformational interconversion (*k*_conf_) relative to that for the wild type or the P177A mutant (as discussed further below). While no similarly pronounced change in the rate constant was observed for the P177A variant, this mutation is significant because subsequent analyses showed that both, this amino-acid replacement and the Y171W variant, redistribute the native energy landscape of AdK (see the section *k*_cat_ versus *k*_M_ and *k*_cat_ versus *K*_D_ compensation). Most of the expected resonances are visible in the NMR spectra of wild-type AdK in complex with saturating concentrations of the natural substrate ADP (Supplementary Fig. 2a). However, many of these resonances are missing in the NMR spectra of the Y171W mutant in complex with ADP because its conformational interchange proceeds at an intermediate rate on the NMR timescale (Supplementary Fig. 2b). In the intermediate exchange regime, the rate of conformational interconversion is on the order of the difference in chemical shifts between exchanging states, resulting in broadened or missing resonances. This observation suggests that the Y171W mutation slowed down the enzyme's conformational dynamics to the point that it entered the intermediate exchange regime at 25 °C. Further support for this hypothesis comes from the finding that NMR experiments using the Y171W mutant under ADP-saturated conditions at a temperature of 50 °C yielded spectra closely resembling those for the ADP-saturated wild type at 25 °C (Supplementary Fig. 2c), indicating that the dynamic properties of the two enzymes are similar at these two temperatures. That is to say, the slowdown of the protein's dynamics induced by the Y171W mutation can be counteracted by raising the temperature. To avoid the problems created by absent resonances in quantitative analysis, we hypothesized that dynamic signatures would also appear in the NMR spectra of AdK variants in complex with the substrate mimic Ap5a (ref. 19). The relevance of using Ap5a when probing conformational dynamics is supported by the results of single-molecule fluorescence spectroscopy experiments, which have shown that both substrate (AMPPNP and AMP)-bound^14^ and Ap5a-bound AdK fluctuate between the closed state and less compact structures^11^. When bound to Ap5a, the NMR spectra of the wild type and P177A variant of AdK feature a single set of resonances because the structural interconversions of their bound states are rapid on the NMR timescale. In sharp contrast, the ^1^H–^15^N heteronuclear single quantum coherence (HSQC) NMR spectrum of Y171W bound to Ap5a shows two sets of resonances at ambient temperature (Fig. 2a and Supplementary Fig. 2d): 61 of its residues give rise to two peaks of roughly equal intensity. These 61 residues are distributed over the entire enzyme (Supplementary Fig. 2d,e), showing that the underlying event is global in nature, and not restricted to the side-chain atoms of Trp171 as seen in the crystal structure (Fig. 1d). It is important to note that the split peaks reflect two different Ap5a-bound states because the enzyme concentration used in the NMR experiments was 500 μM, which is far greater than the *K*_D_ value for the binding of Ap5a to Y171W (*K*_D_=250±60 nM). ZZ-exchange experiments^20^,^21^ are designed to enable quantification of exchange kinetics in cases where two conformational states interchange slowly on the NMR timescale and thus give rise to two distinct peaks, as is the case for Y171W under Ap5a-saturated conditions at 25 °C. In these experiments, exchange kinetics are quantified by monitoring the growth of exchange peaks and the simultaneous depletion of diagonal peaks as shown in Fig. 2b. When this experiment was performed on a sample consisting of Ap5a-bound Y171W, the two states (manifested as dual peaks) were found to be in dynamic equilibrium because they gave rise to exchange cross-peaks (Fig. 2b,c). The rate of conformational interconversion, *k*_conf_, between the two states was determined to be 25±10 s^−1^ at 15 °C (Fig. 2c) using the procedure of Miloushev et *al*.^22^. The data were also analysed following the procedure of Farrow et *al.*^20^ (Supplementary Fig. 2f–j) and both methods resulted in very similar rates of interconversion. A second independent measure of the apparent rate constant, *k*_app_, for Ap5a-induced conformational change of Y171W was obtained using fluorescence stopped-flow experiments, which yielded an Ap5a concentration-independent value of *k*_app_=24±3 s^−1^ at 17 °C (Supplementary Fig. 2k). The initial Ap5a to Y171W binding event occurs within the dead time of the measurement and is therefore silent in the stopped-flow experiment (Supplementary Fig. 2l). Overall, it appears that the Y171W mutation shifts the dynamic response of AdK into a regime where fluctuations in the Ap5a-bound state become slow on the NMR timescale and hence directly observable.

### Characterizing a high-energy state of Ap5a-bound Y171W

**Figure 3:**
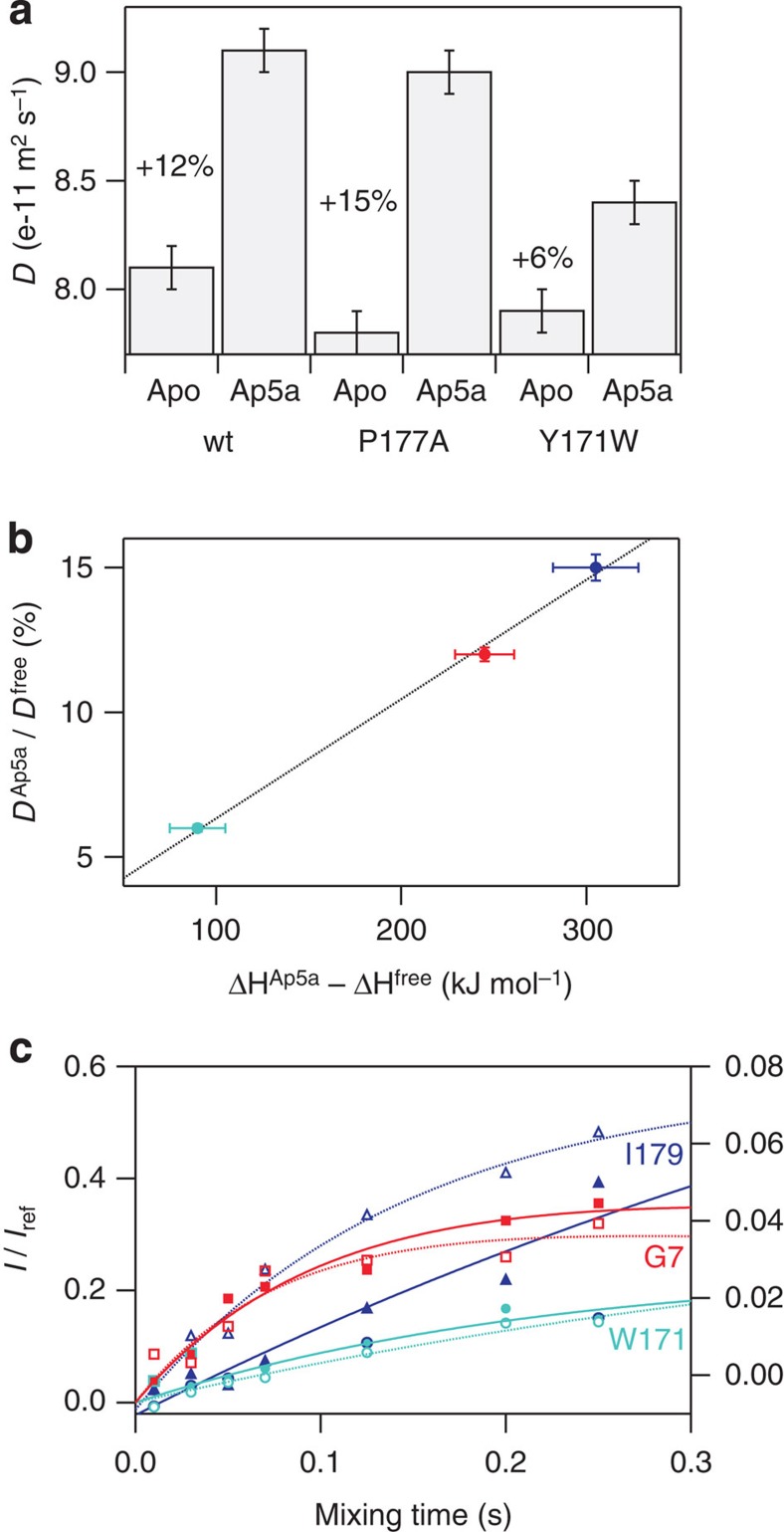
Ap5a-bound Y171W simultaneously populates both open and closed conformations. (**a**) The diffusion coefficients of the wild type, P177A and Y171W AdK variants show that Ap5a-bound Y171W populates both open and closed structures. The bound open conformation corresponds to the high-energy state shown in red in Fig. 1c. (**b**) The relative change in diffusion coefficient between the apo- and Ap5a-bound states correlates with the difference in unfolding enthalpy between these states (wild type in red, P177A in blue and Y171W in cyan). (**c**) Build-up curves for residue-specific proton exchange on the millisecond timescale. Solid and dotted lines correspond to the two exchanging peaks observed for Ap5a-bound Y171W. Residue Ile179 (labelling on right *y* axis) is the only residue shown on the graph whose solvent exchange rate differs significantly between the two states.

What are the underlying structural properties of the two interconverting structural states that give rise to split peaks? To characterize the two states observed in the NMR spectrum of Ap5a-bound Y171W, we exploited the fact that a protein's diffusion coefficient provides an accurate measure of its hydrodynamic dimension^23^. Diffusion experiments were performed on the apo- and Ap5a-bound forms of wild-type AdK to obtain reference data for the open and closed structural ground states, respectively; the more compact closed state was found to diffuse significantly faster than the open apo state (Fig. 3a). To assess the accuracy of these measurements, we also determined the diffusion coefficients of the P177A mutant, which were almost identical to those of the wild type (Fig. 3a). It is important to note that the ratio of the radii of gyration for the open and closed states is consistent with the ratios of the measured hydrodynamic radii for the wild type and AdK P177A (Supplementary Table 1). Therefore, diffusion measurements can be used to characterize the proteins' molecular topology. Strikingly, the diffusion coefficient of Ap5a-bound Y171W is intermediate between that of apo- and Ap5a-bound wild-type AdK (Fig. 3a), and can accurately be modelled as a linear combination of the values for the open and closed states of the wild type in which both have approximately equal weights. Since we observe two sets of resonances in the NMR spectrum of Ap5a-bound Y171W and its diffusion coefficient is equal to the average of those for the open and closed states of the wild type, we assigned the split peaks in its NMR spectrum to two distinct structural states—an Ap5a-bound open conformation and an Ap5a-bound closed conformation. This is the first time that closed and open substrate-bound states have been observed simultaneously. The relationship between the protein's hydrodynamic properties and its molecular structure is underlined by the correlation between the change in the diffusion coefficient comparing apo and Ap5a-bound states and that in the enthalpy of unfolding (Fig. 3b). It was also expected that the solution properties of the Ap5a-bound open and closed states would not only differ with respect to diffusion, and millisecond solvent exchange rates determined using MEXICO^24^ showed that the stabilities of residues close to the substrate-binding pocket (Leu83, Asp110, Val111 and Ile179) differ between the substrate-bound open and closed states (Fig. 3c, Supplementary Fig. 3 and Supplementary Table 2). These results support the assignment of the two sets of NMR peaks to the Ap5a-bound open and closed states because the solvent accessibility of the substrate-binding pocket is expected to increase when the enzyme samples the two conformations. The secondary structure and therefore also the backbone hydrogen bonding patterns of AdK is virtually identical in open and closed states. The amide protons of residues Leu83, Asp110 and Ile179 are hydrogen bonded in both open and closed states, whereas the amide proton of residue Val111 is not engaged in a hydrogen bond in neither of the two states. It thus appears that amide proton exchange is modulated by the combined action of Ap5a interaction and the structural interconversion between Ap5a-bound open and closed states.

The results presented above raise an important question: is the Ap5a-bound open structure observed for the Y171W mutant also populated in wild-type AdK? This was answered by examining the rate of interconversion between the structural states of Ap5a-bound Y171W at different temperatures. Raising the temperature from 10 to 50 °C caused peaks that are split in the ^1^H–^15^N HSQC spectra of Y171W at low temperatures to merge into single peaks, showing the transition in the exchange regime from slow (on the NMR timescale) to fast as the temperature increases (Supplementary Fig. 4). Both enzyme variants thus behave similarly at high temperature, and we can conclude that the chemical shifts observed under Ap5a-saturated conditions represent an average of the shifts for the open and closed Ap5a-bound states.

The performed line shape analysis provided rates of conformational exchange of 10 and 20 s^−1^ at 10 and 20 °C (Supplementary Fig. 4), respectively, in good agreement with the ZZ-exchange experiment (Fig. 2c and Supplementary Fig. 2f–j).

### *k*_cat_ versus *K*_M_ and *k*_cat_ versus *K*_D_ compensation

Since AdK is rate limited by the rate constant for the opening of its substrate-binding subdomains (*k*_open_), its binding affinities (quantified in terms of *K*_M_ or *K*_D_) and *k*_cat_ values are both modulated in response to changes in *k*_open_ (Fig. 1c). The reason is that *k*_cat_ is equal to *k*_open_ and that binding affinities depend on the statistical weight of the closed state that is governed by *k*_open_ and *k*_close_. The dependency of *k*_cat_ and *K*_M_ on the equilibrium constant between open and closed states has been reported previously. It was found that perturbations that affect the native free-energy landscape of AdK give rise to ‘*k*_cat_ versus *K*_M_ compensation' whereby *k*_cat_ and *K*_M_ are anti-correlated such that the specificity constant (*k*_cat_/*K*_M_) is silent to the perturbation^13^. Since a similarly strong correlation was observed between the *k*_cat_ and *K*_M_ values for the AdK variants examined in this work (Supplementary Fig. 5a), we conclude that the effect of the Y171W and P177A replacements merely perturbs the functional energy landscape of wild-type AdK, and that the conclusions drawn from experiments on Y171W are relevant to the functional cycle of wild-type AdK. Furthermore, the observation of *k*_cat_ versus *K*_D_ compensation for Ap5a binding (Supplementary Fig. 5b) is important because it shows that the structure and properties of this substrate analogue adequately mimic those of the enzyme's natural substrates and products (AMP, ATP and ADP) during its functional catalytic cycle. Interestingly, we observed a hyperbolic temperature dependency in the *K*_D_ values for the wild type, P177A and Y171W variants, with the binding affinity peaking at the growth temperature of *Escherichia coli* (Supplementary Fig. 5c). It thus appears that the dynamic properties underlying the enzyme's binding affinity are optimized for functionality at 37 °C, which substantiates the inference that enzymes' dynamics develop under evolutionary constraints^25^.

## Discussion

**Figure 4:**
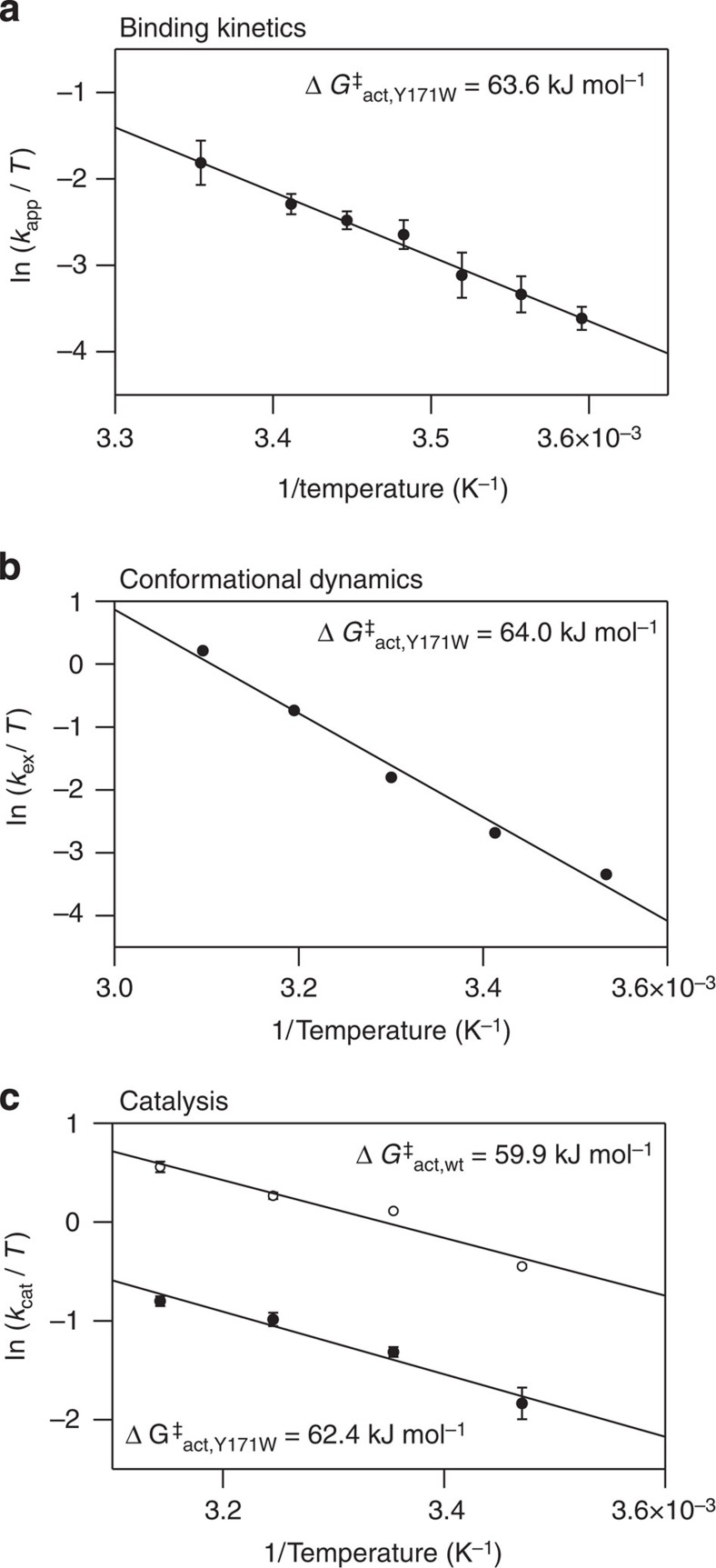
Activation barriers for conformational dynamics and catalysis. Barrier heights were determined at 25 °C from Eyring–Polanyi analyses by monitoring (**a**) apparent rate constants for Ap5a binding to Y171W based on fluorescence stopped-flow experiments, which yielded kinetic parameters of Δ*H*^sf^=(62.1±2.8) kJ mol^−1^ and Δ*S*^sf^=−(4.7±0.2) J mol^−1^ K^−1^; and (**b**) conformational exchange dynamics for residue Thr89 of Y171W based on NMR line shape analysis. Eyring–Polanyi analysis in this case yields kinetic parameters of Δ*H*^LS^=(68.6±4.7) kJ mol^−1^ and Δ*S*^LS^=(15.3±1.1) J mol^−1^ K^−1^. (**c**) Catalytic turnover values for the wild type (open circles) and Y171W (closed circles) AdK variants determined using a coupled assay^35^. Eyring–Polanyi analysis yields kinetic parameters of Δ*H*^act^_wt_=(24.1±4.2) kJ mol^−1^ and Δ*S*^act^=−(120±20) J mol^−1^ K^−1^ for wild type, and Δ*H*^act^_Y171W_=(26.6±3.3) kJ mol^−1^ and Δ*S*^act^_Y171W_=−(120±20) J mol^−1 ^K^−1^ for Y171W variant.

**Figure 5:**
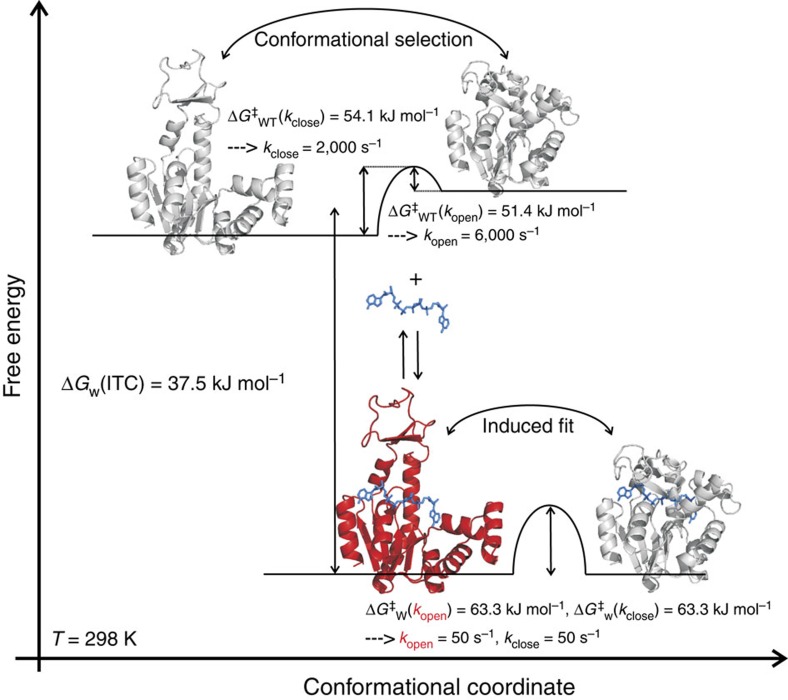
The high-energy state is crucial for the catalytic mechanism of AdK. The state shown in red corresponds to the directly observed high-energy state of Y171W and is modelled from known structures (1AKE and 4AKE). This state completes the description of conformations required for AdK catalysis. Relative ground state free energies and activation barriers, Δ*G*^‡^, are indicated for apo-11 and substrate-bound AdK. The binding of Ap5a to Y171W lowers the free energy of the system by 37.5 kJ mol^−1^. Kinetic rate constants are displayed for the dynamic interconversion between structural states. Taken together, the data show that population of closed AdK conformations is accomplished through conformational selection^29^ in the apo state and induced fit in the presence of substrate^27^. Subscripts ‘WT' and ‘W' correspond to wild type and Y171W, respectively. Although no direct high-resolution structural data exist for the closed apo structure, indirect evidence from ensemble^49^ and single-molecule^11^ fluorescence resonance energy transfer experiments and chemical shifts^13^ points towards that compact ‘closed-like' structures are sampled by AdK in the absence of substrate.

Protein engineering has enabled us to directly observe, characterize and manipulate a functionally indispensable high-energy state of AdK. This is a significant development because while such states are known to be essential for enzymatic catalysis^4^,^6^,^9^, it was previously impossible to study them directly. Our approach is based on specific amino-acid replacements in a region that is known to affect the allosteric opening and closing reaction of AdK. This confined spatial hotspot for manipulation of an enzyme's dynamics is analogous to that found in maltose binding protein, where the replacement of two specific residues markedly change the enzyme's ligand binding affinity and conformational dynamics^26^. The high-energy AdK state corresponds to an open substrate-bound conformation that is in equilibrium with a stable substrate-bound ground state populating a closed structure. With the experimental set-up, it is not possible to determine whether the domain motions are correlated, anti-correlated or uncorrelated. There must be a structural reason for the change in dynamics that is observed in the Y1711W variant and that enabled the direct detection of the high-energy state. In this particular case, the altered dynamics likely depend on the two different side-chain conformations of Y171W in the Ap5a-bound state. In the crystal structure of Ap5a-bound Y171W, the enzyme is fully closed, whereas NMR measurements show that Ap5a-bound Y171W exchanges between open and closed states in solution. Hence, it appears that one of the side-chain conformations observed in the crystal structure triggers opening of the enzyme in solution. We were able to determine the molecular topology of the high-energy state and also to directly quantify the kinetic parameters of the conformational dynamics connecting it to the stable ground state. A key prediction of the catalytic model developed for AdK, which suggests that conformational dynamics are rate limiting in the catalytic cycle, is that free-energy barriers determined by measuring the rate of conformational interconversion should converge with those determined by examining the enzyme's catalytic turnover. This prediction was confirmed: the barrier height estimated by analysing the temperature dependence of the enzyme's conformational dynamics (Fig. 4a,b; 63.8±0.2 kJ mol^−1^) is in good agreement with that determined on the basis of measured enzyme activity (Fig. 4c; 62.4±3.3 kJ mol^−1^). From a mechanistic standpoint, these results resolve the long-standing question about whether the induced fit^27^ or conformational selection^28^,^29^ models are most appropriate for describing the binding of substrates to enzymes. The hallmark of the conformational selection model is that active conformations are sampled even in the absence of the substrate, and that substrate addition merely redistributes the relative population of inactive versus active states. It has previously been suggested that substrate-free AdK samples closed-like structures. In a complete description of ligand binding to AdK through competing conformational selection or induced fit models^30^ it is assumed that the closed apo structure is a binding competent state. This has however not been shown experimentally and it is possible that the substrate-binding sites are occluded due to steric hindrance in the apo-closed structure^31^. Our kinetic stopped-flow data show that AdK is using an induced fit mechanism for closure following the initial binding event, thus the conformational change occurs after ligand binding. Hence, the conformational change occurs by two different mechanisms in apo- and substrate-bound states. In absence of substrate domain closure occurs by conformational selection, whereas induced fit is utilized in the presence of substrate. However, in a competition between induced fit and conformational selection pathways, the flux through either path is determined by intracellular substrate concentrations and all contributing rate constants as discussed in ref. 30. The rate constants for the enzyme's conformational dynamics appear to have been determined by natural selection because its substrate-binding affinity (which is governed by its conformational dynamics) is highest at the preferred growth temperature of *E. coli*. The direct NMR-based characterization of both the structure and dynamics of a high-energy state that is functionally relevant to AdK catalysis enables us to establish a detailed reaction scheme based on the conformational states that constitute the structural framework for AdK catalysis (Fig. 5).

The *k*_cat_ versus *K*_D_ compensation discovered here is adding additional insights compared to the *k*_cat_ versus *K*_M_ compensation discovered previously for AdK^13^. The compensatory effect on activity and binding affinities is phenomenologically related to results reported for the Dbl homology domain of the guanine exchange factor Vav1 where it was found that the specificity constant (*k*_cat_/*K*_M_) depends linearly on the population of a high-energy state^32^. A related dependency on the specificity constant on the population of a high-energy state has also been reported for phosphorylation of phospholamban by cAMP-dependent protein kinase A^33^. As a final note, we believe that our results indicate that there is great potential for exploiting the rational tuning of conformational dynamics in the *de novo* design of enzymes.

## Methods

### Sample preparation

Wild-type AdK, P177A and Y171W were overexpressed in BL21(DE3) cells from plasmids controlled by the AdK promotor. Cells were harvested by centrifugation and resuspended in a buffer consisting of 50 mM Tris, pH 7.5. After a freeze-thaw cycle, the cells were lysed by sonication and centrifuged to remove cell debris. Purification of supernatants from sonication was accomplished with Blue Sepharose using a linear NaCl gradient for elution. Pooled fractions containing AdK were subjected to size-exclusion chromatography in a buffer consisting of 30 mM MOPS and 50 mM NaCl, pH 7.0, and fractions containing AdK were pooled, concentrated and stored at −20 °C. All protein concentrations of wild type and P177A were determined using an extinction coefficient of 10,430 M^−1^ cm^−1^ and for Y171W of 14,440 M^−1^ cm^−1^ at *λ*=280 nm. Nucleotide concentrations were determined using an extinction coefficient of 30,000 M^−1^ cm^−1^ (Ap5a) and 15,400 M^−1^ cm^−1^ (ADP) at *λ*=260 nm.

### Site-specific mutagenesis

Primer sequences for the P177A and Y171W mutants were ordered from Eurofins MWG Operon. The mutants were made using the QuikChange method (Stratagene), expressed in a pEAK-91 vector^34^ and verified by DNA sequencing (Eurofins MWG Operon).

### Fluorescence kinetic stopped flow

A sequential mixing stopped-flow spectrometer (Chirascan, Applied Photophysics) was used for all kinetic experiments. The reaction kinetics of Ap5a binding to Y171W was observed by the change in tryptophan fluorescence intensity using a cutoff filter of 340 nm after excitation at a wavelength of 295 nm. All experiments were carried out in 30 mM MOPS and 50 mM NaCl, pH 7.0. Mixing experiments were performed at 5, 8, 11, 14, 17, 20 and 25 °C. The temperature of the whole mixing device was maintained with a circulating water bath. Binding reactions were initiated by a rapid 1:10 mixing ratio of different concentrations of Ap5a with a solution containing 15 μM Y171W. An additional control mixing experiment was performed to exclude any effect of Ap5a on the buffer. No influence to fluorescence emission was observed. Kinetic time traces were collected at least 10 times under identical conditions and were averaged for the fitting procedure according to equation (1):


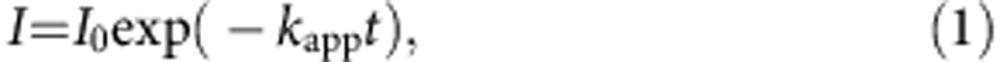


where *I* is the fluorescence emission intensity and *k*_app_ is the apparent rate constant for the binding event. Temperature-dependent apparent rate constants were plotted against the inverse absolute temperature and fitted using the linear form of the Eyring–Polanyi equation (2):


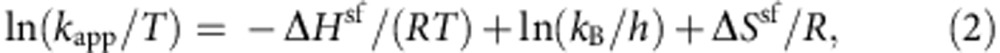


with the absolute temperature *T*, Boltzmann constant *k*_B_, Planck constant *h*, the change in enthalpy Δ*H*^sf^ and the change in entropy Δ*S*^sf^ caused by Ap5a interaction to Y171W. The abbreviation ‘sf' stands for stopped flow. Using the Gibbs–Helmholtz equation (3) leads to the determination of the activation barrier, ΔG^‡^_act_, at a specific temperature *T:*


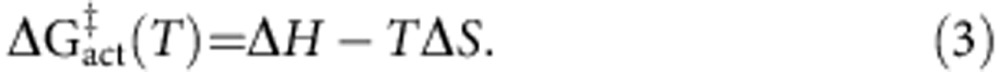


### Kinetic assay probing enzyme activity

Enzyme kinetic parameters for ATP turnover in the direction of ADP formation were quantified for wild-type AdK and Y171W at 15, 25, 35 and 45 °C and for P177A at 25 °C using a coupled spectroscopic assay^35^ on a Varian Cary 50 UV-Visible Spectrophotometer. Practically, the ATP concentration was varied and ATP was added together with 10 μl of protein to a solution containing 100 mM Tris, 80 mM KCl, 0.2 mM NADH, 0.4 mM PEP, 0.3 mM AMP and 2 mM MgCl_2_ at pH 7.5 giving a final volume of 471 μl. Monitoring the time-dependent change of the absorption value at a wavelength of 340 nm leads finally to the velocity of the reaction. This reaction velocity, *v*, was plotted against ATP concentration, *c*_ATP_, and using Michaelis–Menten's relation (equation (4)) enables extraction of maximal turnover rate, *k*_cat_, and ATP affinity, *K*_M_, for the used spectroscopic assay:


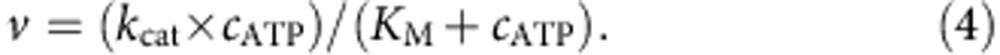


Eyring–Polanyi analysis was performed as described for fluorescence kinetic stopped flow.

### Structure determination by X-ray crystallography

Crystallization. Before crystallization, the proteins were stored in buffer containing 50 mM NaCl and 30 mM MOPS adjusted to pH 7.0. The protein concentrations of Y171W and P177A variants were adjusted to 12 and 22 mg ml^−1^, respectively. The concentrations were maintained in the presence of the inhibitor P1,P5-di(adenosine-5')pentaphosphate, Ap5a. Sitting drop crystallization set-ups in 96-well MRC two-well crystallization plates (Molecular Dimensions) were carried out with a Mosquito Nanodrop pipetting robot (TTP Labtech). Initial crystallization conditions were obtained from Crystal Screen and Crystal Screen 2 (Hampton Research) and manually optimized in 24-well Linbro plates (Hampton Research) using the hanging-drop vapour diffusion method. The final drops contained 2 μl of Y171W or Y171W/Ap5a solution (five-time stoichiometric excess) mixed with 1 μl of reservoir solution and equilibrated over 1 ml reservoir buffer. Owing to its higher initial protein concentration, only 1 μl of the P177A or P177A/Ap5a solution (five-time stoichiometric excess) was mixed with 1 μl of precipitation buffer. The crystals diffracting best were obtained at 18 °C and with the following solutions: Y171W in 0.1 M Tris (pH 8.0), 0.2 M NaOAc and 28% PEG 4 K; Y171W/Ap5a in 0.1 M sodium citrate (pH 6.0), 0.2 M ammonium acetate and 28% PEG 4 K; P177A in 0.1 M Tris (pH 8.5), 0.2 M MgCl_2_ and 30% PEG 4 K; and P177A/Ap5a in 0.1 M Tris (pH 8.5), 0.2 M ammonium acetate and 30% PEG 4 K.

Data collection, structure determination and refinement. Crystals were equilibrated in cryo-protectant buffer adjusted to 20% (v/v) glycerol in the respective reservoir solution before flash-cooling to 100 K. All X-ray diffraction data sets were collected at a wavelength of 0.15418, nm (Cu-Kα radiation) on an in-house X8-Proteum system equipped with a MicroStar-H X-ray generator (Bruker AXS). Diffraction data were integrated with SAINT and scaled using SADAPS (Bruker AXS programme suite). Data collection statistics are listed in Table 1.

All four AdK structures were determined by molecular replacement with the programme PHASER from the PHENIX programme suite^36^ using the *E. coli* ApK apo and holo structures, pdb code 4AKE (ref. 37) and 1AKE (ref. 38) as search models. The electron density maps were well defined and allowed the atomic models to be built using the programme Coot^39^ and refined with PHENIX Refine^36^. The AdK structures were superimposed and analysed with ICM-Pro (Molsoft LLC), which was also used to prepare the structural figures. The atomic coordinates and the structure factors have been deposited within the Protein Data Bank^40^ (PDB codes 4X8M for Y171W-apo, 4X8O for Y171W-holo, 4X8H for P177A-apo and 4X8L for P177A-holo).

### CD spectroscopy

Protein unfolding of the apo and Ap5a-bound wild type, P177A and Y171W AdK proteins was monitored by measuring the ellipticity at 220 nm using a 1-mm path length quartz cuvette on a Jasco J-810 spectropolarimeter. The proteins were stored in 30 mM MOPS and 50 mM NaCl, pH 7.0, and concentrations for the CD experiments ranged between 5 and 10 μM. Ap5a was titrated to a five-time stoichiometric excess. Equation (5) was used to get the midpoint, *T*_m_, and the van't Hoff enthalpy of unfolding, Δ*H*_vH_, at *T*_m_.


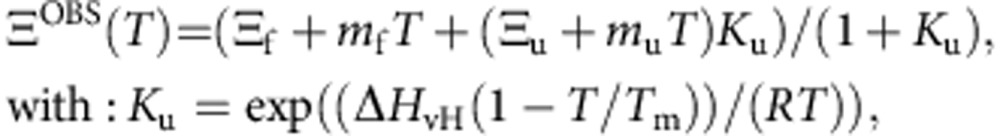


where Ξ^OBS^ is the temperature-dependent observed ellipticity at 220 nm, *m*_f_, *m*_u_, Ξ_f_ and Ξ_u_ are the slopes and intercepts of the baselines related to folded and unfolded conditions, *K*_u_ is the equilibrium constant in direction of unfolding, *T* is the absolute temperature and *R* represents the gas constant.

### NMR spectroscopy

All NMR experiments were conducted at a Bruker Avance III HD 850 MHz spectrometer equipped with a z-gradient cryogenic probe using 30 mM MOPS and 50 mM NaCl, pH 7.0, and 5% D_2_O (v/v) as sample buffer. All two-dimensional (2D) and 3D NMR data sets were processed with NMRPipe^41^ and analysed in NMRView^42^.

*Assignment experiments*. Backbone ^15^N, ^1^H chemical shifts have been previously published for wild-type AdK for both apo-43 and Ap5a-bound conditions^44^, and were verified using ^1^H–^15^N NOESY-HSQC spectroscopy. Of 204 non-prolyl residues, 198 (apo) and 195 (Ap5a bound) could be assigned. Backbone ^15^N, ^1^H chemical shifts for P177A and Y171W in the apo- and Ap5a-bound states were obtained by comparison with the wild-type chemical shifts and acquisition of ^1^H–^15^N NOESY-HSQC spectra. For P177A, 198 (apo) and 181 (Ap5a bound) could be assigned out of 205 non-prolyl residues. For Y171W, 200/204 (apo) and 265 (Ap5a bound) out of 204 non-prolyl residues could be assigned.

The assigned chemical shifts have been deposited within the BMRB database for P177A-apo (accession number 25353), Y171W-apo (accession number 25357), P177A-holo (accession number 25360), Y171W-holo data set a (accession number 25361) and Y171W-holo data set b (accession number 25362).

Weighted differences in chemical shifts Δ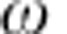 is calculated by using equation (6)


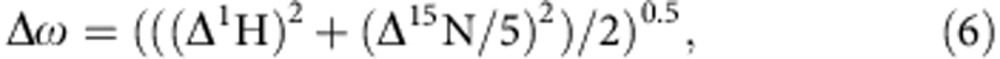


where Δ^1^H is the change in proton and Δ^15^N is the change in nitrogen dimension, respectively.

*Diffusion experiments*. NMR diffusion spectra were recorded using pulsed field bipolar gradient stimulated echo experiments^45^ at 25 °C. For each diffusion profile, 21 different gradient strengths *G* were used for 6 ms along the *z* axis followed by a 100 μs recovery delay. The diffusion of AdK molecules was allowed to proceed for 100 ms to ensure a protein signal intensity decay of ∼90% at maximum gradient strength. The calibration of *G* was performed by a standard protocol^46^. For error estimation, four different gradient strengths were repeated (relative gradient strengths of 1, 10, 40 and 70%). The measured ^1^H NMR spectra were integrated within the aliphatic signal region (−0.5... 2.5 p.p.m.) and fitted to equation (7):


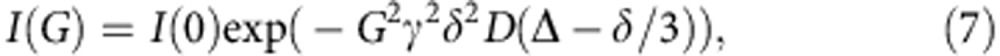


where *γ* is the gyromagnetic ratio, *δ* is the gradient length, Δ is the diffusion time and *D* is the calculated diffusion coefficient^45^. Water suppression was achieved by presaturation and WATERGATE. Spectra were processed and analysed by Topspin 3.2.

*Line shape analysis*. For line shape analysis, ^15^N slices were extracted from 2D ^1^H–^15^N HSQC spectra recorded at 10, 20, 30, 40 and 50 °C for Ap5a-bound Y171W. Subsequently, a two-component Lorentzian function was fitted to individual 1D ^15^N spectra to obtain intensity offsets, *y*_1_ and *y*_2_, chemical shift offsets, *δ*_1_ and *δ*_2_, peak amplitudes, *A*_1_ and *A*_2_ and line widths, *w*_1_ and *w*_2_:


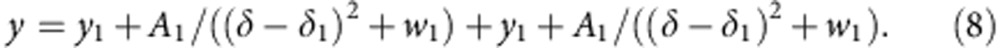


The rate of conformational exchange, *k*_ex_, between the states 1 and 2 was introduced by the McConnell equations^47^. Populations and chemical shift offsets for both states were used from Lorentzian regression as discussed above during simulation of ^15^N NMR line shape.

Eyring–Polanyi analysis was performed as described for fluorescence kinetic stopped flow.

*ZZ-exchange spectroscopy*. ZZ-exchange experiments for Ap5a-bound Y171W were set-up according to Farrow *et al.*^20^ and run at 15 °C with mixing times, *t*_mix_, of 20, 30, 40 and 100 ms. The rate constant of conformational interconversion, *k*_conf_, was calculated using the procedure of Miloushev et *al*.^22^:


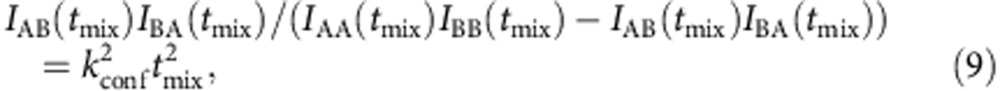


where *I*_AA_, *I*_BB_ are auto peak and *I*_AB_, *I*_BA_ are cross-peak intensities for individual residues.

*MEXICO*. Fast millisecond proton exchange was determined from 2D ^1^H–^15^N HSQC spectra using a modified MEXICO^24^ pulse sequence applying 10, 30, 50, 70, 125, 200 and 250 ms mixing time delays. Cross-peak intensities were referenced to a conventional 2D ^1^H–^15^N HSQC and analysed according to Hofmann *et al.*^48^.

### Isothermal titration calorimetry

Isothermal titration calorimetry experiments were carried out at 15, 25, 35 and 45 °C using MicroCal Auto-iTC200 isothermal titration calorimeter (GE Healthcare). The reference cell was filled with sample buffer (30 mM MOPS, 50 mM NaCl, pH 7.0). Protein solutions (380 μl) were filled into the sample cell and titrated with Ap5a loaded in the syringe (100 μl). The following set-up was used: reference power of 4 μcal s^−1^, initial delay of 200 s, stirring speed of 400 r.p.m., spacing of 150 s, filter of 5 s, injection of 0.3 μl (first injection) or 1.5 μl (following injections) and low-feedback mode.

Particular protein/Ap5a concentration ratios were 60 μM/541 μM, 90 μM/541 μM and 120 μM/1,083 μM for Y171W, 75 μM/541 μM for wild type and 97 μM/541 μM for P177A titration at 25 °C; 65 μM/1,109 μM for all performed experiments at 15 °C and 35 °C; 65 μM/650 μM for Y171W and wild type; and 45 μM /555 μM for P177A at 45 °C. Control experiments (injection of Ap5a into buffer solution) did not show any change in enthalpy. Raw data were collected, corrected for ligand heats of dilution and integrated using the MicroCal Origin software. Data sets were fitted by using a single-site binding model to yield the binding constant, *K*_D_, the apparent enthalpy of binding, Δ*H*, and the stoichiometry of binding, *n*.

## Additional information

**Accession codes:** The X-ray structures of AdK Y171W-apo, AdK P177A-apo, AdK Y171W-holo and AdK P177A-holo have been deposited to the Protein Data Bank under accession codes 4X8M, 4X8H, 4X8O and 4X8L, respectively. The assigned NMR chemical shifts have been deposited within the BMRB database for P177A-apo (accession number 25353), Y171W-apo (accession number 25357), P177A-holo (accession number 25360), Y171W-holo data set a (accession number 25361) and Y171W-holo dataset b (accession number 25362).

**How to cite this article**: Kovermann, M. *et al.* Structural basis for catalytically restrictive dynamics of a high-energy enzyme state. *Nat. Commun.* 6:7644 doi: 10.1038/ncomms8644 (2015).

## Supplementary Material

Supplementary Information Supplementary Figures 1-5, Supplementary Tables 1-2 and Supplementary References

## Figures and Tables

**Table 1:** X-ray data collection and refinement statistics.

	**P177A-Holo**	**P177A-Apo**	**Y171W-Holo**	**Y171W-Apo**
*Data collection*				
Space group	P2_1_2_1_2_1_	C2	P2_1_2_1_2_1_	C2
Cell dimensions				
*a*, *b*, *c* (Å)	82.57, 72.67, 79.31	135.42, 31.53, 52.75	82.26, 72.65, 79.48	123.57, 31.62, 63.08
*α*, *β*, *γ* (°)	90.0, 90.0, 90.0	90.0, 110.65, 90.0	90.0, 90.0, 90.0	90.0, 114.42, 90.0
Resolution (Å)	27.3–1.70 (1.74–1.70)*	33.6–2.5 (2.59–2.50)	41.2–2.10 (2.18–2.10)	33.8–2.60 (2.67–2.60)
*R*_merge_	0.061 (0.267)	0.139 (0.345)	0.091 (0.363)	0.074 (0.275)
*I*/σ*I*	19.5 (5.0)	16.57 (5.3)	32.0 (6.48)	26.0 (3.7)
Completeness (%)	99.4 (96.8)	98.5 (89.0)	99.7 (98.1)	97.5 (82.3)
Redundancy	7.1 (4.5)	5.22 (4.2)	14.79 (4.88)	4.2 (2.4)
				
*Refinement*				
Resolution (Å)	27.0–1.70	33.6–2.5	41.2–2.10	33.8–2.60
No. of reflections	52,850 (3,261)	7,465 (849)	28,378 (2,747)	7,007 (640)
*R*_work_	0.177 (0.310)	0.191 (0.268)	0.177 (0.216)	0.246 (0.303)
R_free_	0.211 (0.383)	0.289 (0.347)	0.239 (0.286)	0.309 (0.361)
No. of atoms				
Protein	3,406	1,654	3,431	1,652
Ligand/ion	118	—	117	—
Water	842	150	337	36
*B*-factors				
Protein	13.4	42.9	14.4	51.2
Ligand/ion	9.3	—	10.2	—
Water	25.2	43.2	17.4	35.9
r.m.s.d.				
Bond lengths (Å)	0.003	0.003	0.004	0.003
Bond angles (°)	0.872	0.64	0.856	0.650

r.m.s.d., root mean squared deviation.

^*^One crystal was used for data collection. Values in parentheses are for highest-resolution shell.
