# Supplementary material for: Structural basis for catalytically restrictive dynamics of a high-energy enzyme state
Source: Nat Commun. 2015 Jul 3;6:7644. doi: 10.1038/ncomms8644 (PMC4506515; doi:10.1038/ncomms8644)
Supplement: Supplementary Information — Supplementary Figures 1-5, Supplementary Tables 1-2 and Supplementary References [file ncomms8644-s1.pdf]

Supplementary Material

Structural basis for catalytically restrictive dynamics of a high-energy enzyme state

Michael Kovermann, Jörgen Ådén, Christin Grundström, A. Elisabeth Sauer-Eriksson, Uwe H. Sauer & Magnus Wolf-Watz

Department of Chemistry, Umeå University, 90187 Umeå, Sweden

*Correspondence to: magnus.wolf-watz@chem.umu.se

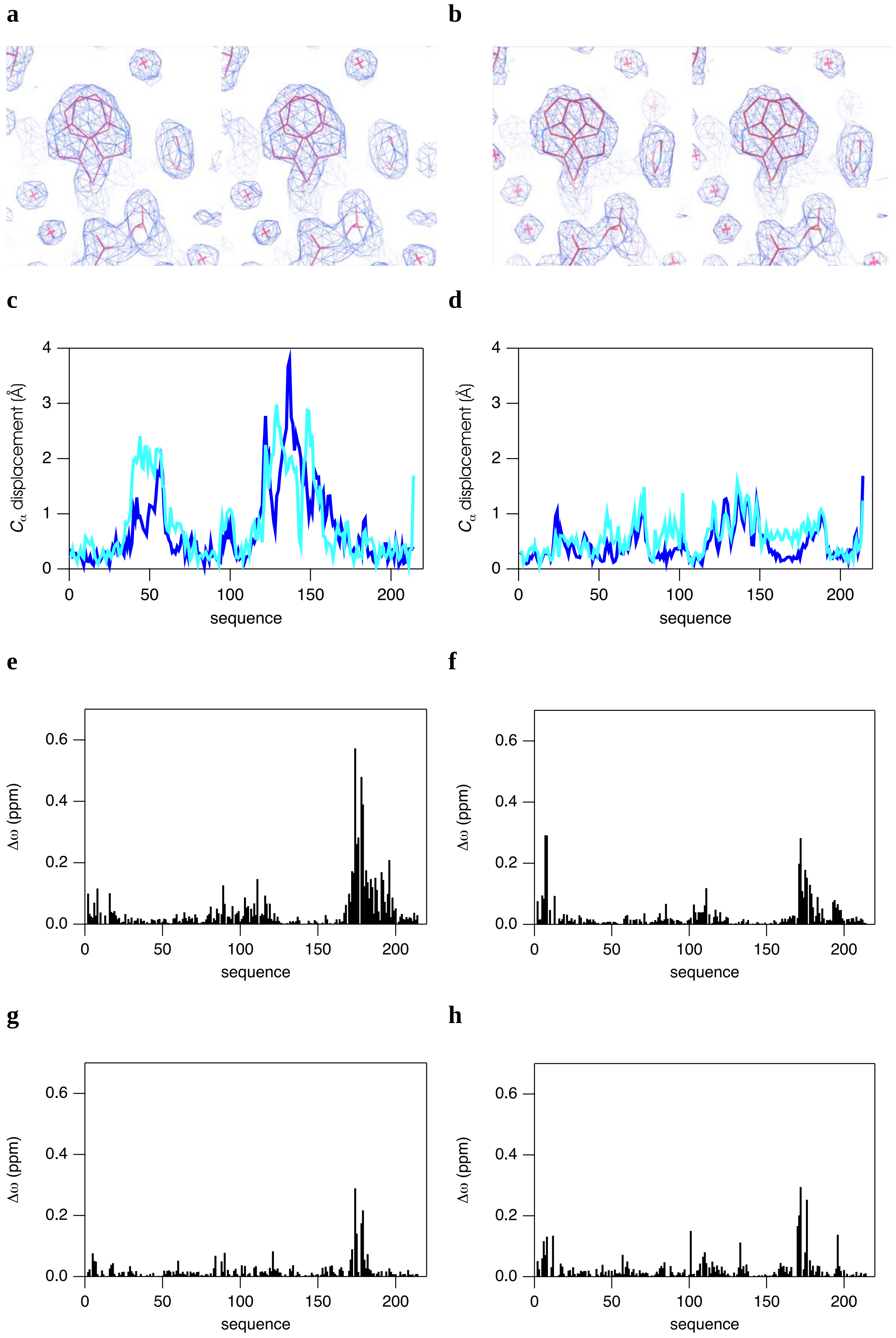
a
b
c
d
e
f
g
h
C_α displacement (Å)
sequence
Δω (ppm)
0
50
100
150
200
0.0
0.2
0.4
0.6
1
2
3
4

i

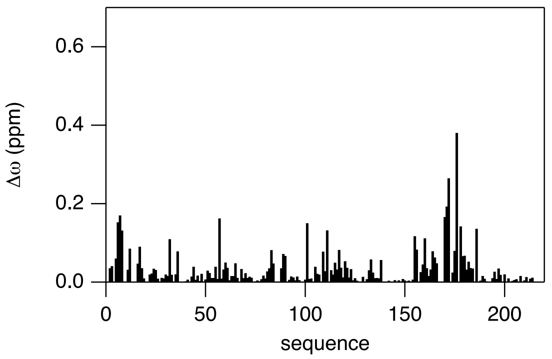

j

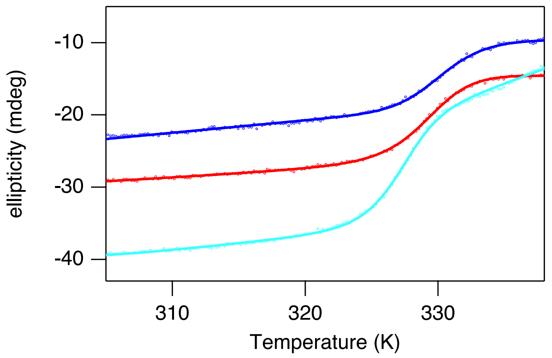

k

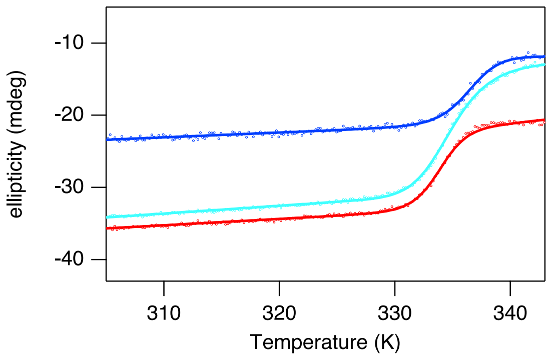

Supplementary Figure 1 Structural features of AdK mutants. (**a**) Quality of the electron density map for the Trp171 residue in AdK Y171W structure bound to Ap5a (4X8O). Lightblue mesh is 2mFo – DFc density contoured at 1.0 rmsd level (Coot map levl = 0.4 electrons/Å^3) for the Trp171 side chain of molecule A in the asymmetric unit. (**b**) The same as in (**a**) but for the Trp171 side chain of molecule B. (**c**) Backbone C_α displacements of Y171W 4X8M, cyan) and P177A 4X8H, blue) with respect to wild type AdK in the apo state (4AKE). (**d**) Backbone C_α displacements of the Ap5a-bound state of Y171W (4X8O) and P177A 4X8L) compared to wild type AdK (1AKE). Color code as in (**c**). (**e**) Weighted chemical shift differences for P177A relative to apo wild type AdK. (**f**) Weighted change of chemical shifts for Y171W relative to wild type AdK in the apo state. (**g**) Weighted change of chemical shifts for P177A relative to wild type AdK bound to Ap5a. (**h**) Weighted change of chemical shifts for signal set "a" of Y171W relative to Ap5a-bound wild type AdK. (**i**) Weighted change of chemical shifts for signal set "b" of Y171W relative to wild type in the Ap5a-bound state. (**j**) Change of molar ellipticity at 220 nm for wild type AdK (red), P177A (blue) and Y171W (cyan) in the apo state. Thermodynamic parameters for the temperature unfolding reaction are presented in **Fig. 1e**. (**k**) Change of molar ellipticity at 220 nm for wild type (red), P177A (blue) and Y171W (cyan) under conditions where Ap5a is bound. Thermodynamic parameters for temperature unfolding reaction are presented in **Fig. 1e**.

a

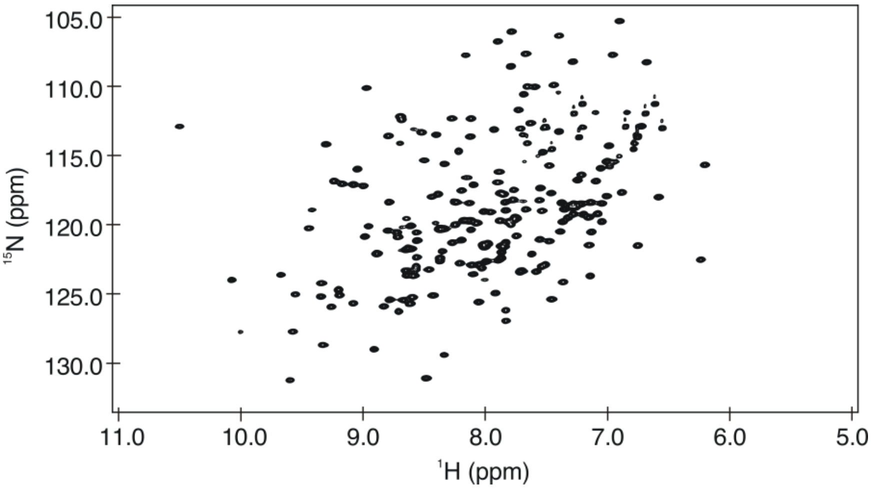
105.0
110.0
115.0
120.0
125.0
130.0
^{15}N (ppm)
11.0
10.0
9.0
8.0
7.0
6.0
5.0
^{1}H (ppm)

b

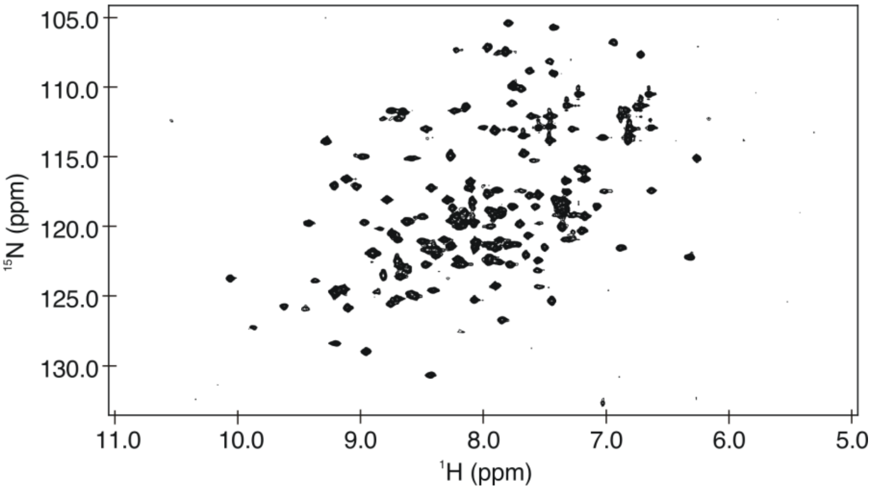
105.0
110.0
115.0
120.0
125.0
130.0
^{15}N (ppm)
11.0
10.0
9.0
8.0
7.0
6.0
5.0
^{1}H (ppm)

c

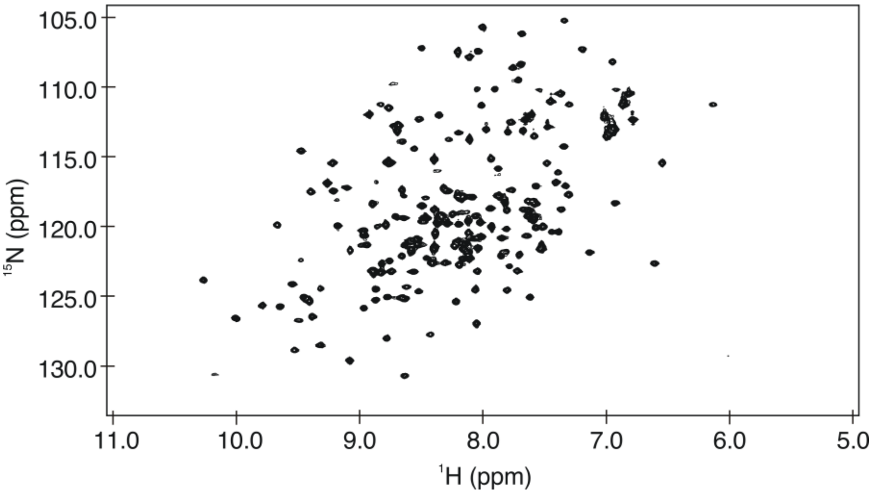
105.0
110.0
115.0
120.0
125.0
130.0
^{15}N (ppm)
11.0
10.0
9.0
8.0
7.0
6.0
5.0
^{1}H (ppm)

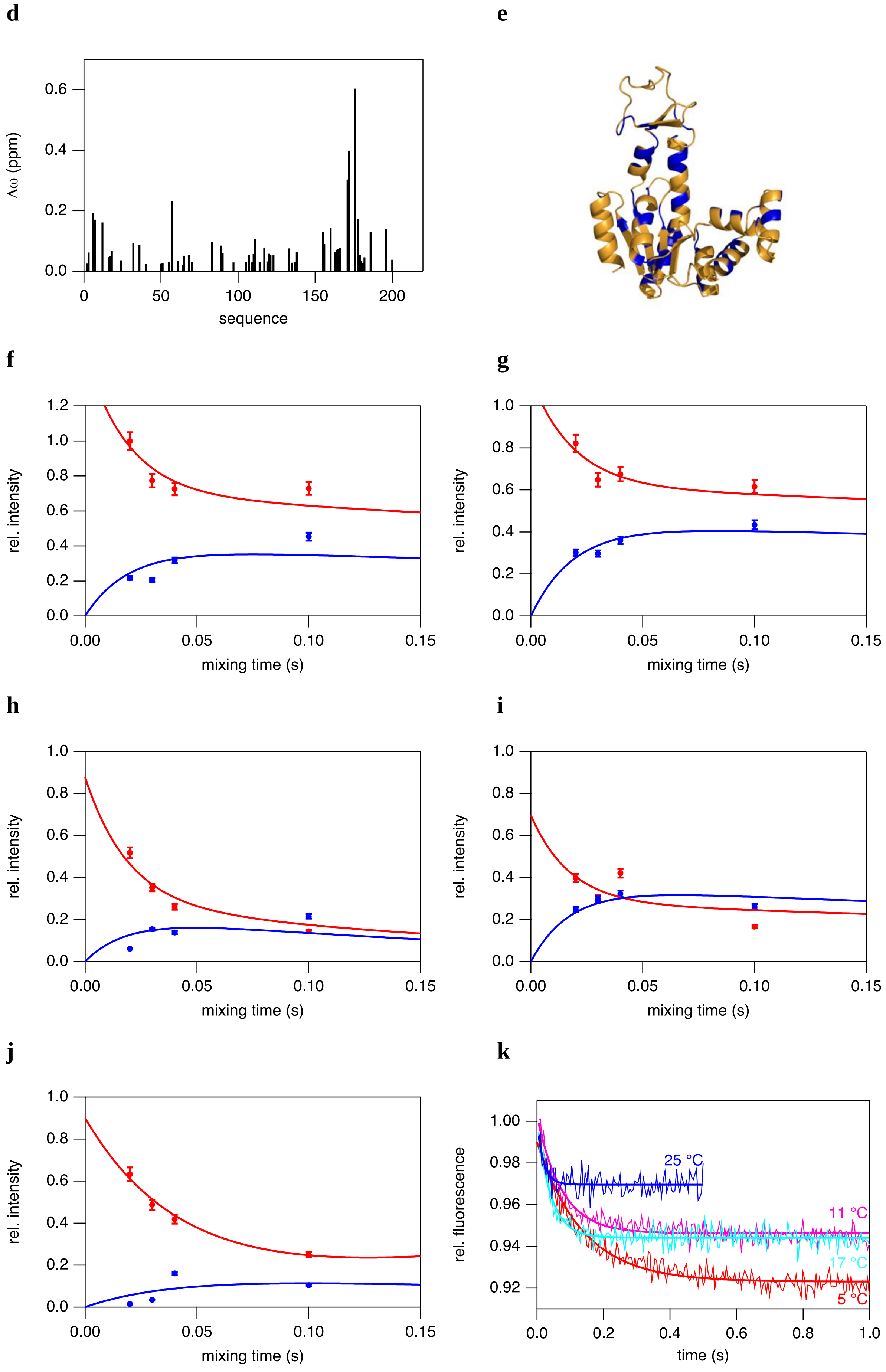

d
Δω (ppm)
0.6
0.4
0.2
0.0
0
50
100
150
200
sequence
e
f
rel. intensity
1.2
1.0
0.8
0.6
0.4
0.2
0.0
0.00
0.05
0.10
0.15
mixing time (s)
g
rel. intensity
mixing time (s)
h
rel. intensity
mixing time (s)
i
rel. intensity
mixing time (s)
j
rel. intensity
mixing time (s)
k
rel. fluorescence
1.00
0.98
0.96
0.94
0.92
25 °C
11 °C
17 °C
5 °C
0.0
0.2
0.4
0.6
0.8
1.0
time (s)

l

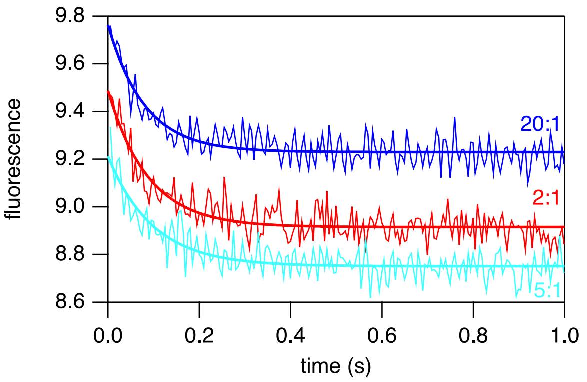

Supplementary Figure 2 ADP and Ap5a interaction with AdK. (**a**) ^{1}H-^{15}N HSQC for ADP saturated wild type AdK acquired at 25 °C. (**b**) ^{1}H-^{15}N HSQC for ADP saturated Y171W acquired at 25 °C. (**c**) ^{1}H-^{15}N HSQC for ADP saturated Y171W acquired at 50 °C. (**d**) Weighted change of ^{1}H and ^{15}N chemical shifts between signal set "a" and signal set "b" of Y171W in the Ap5a-bound state. (**e**) All assigned residues for Y171W in the Ap5a-bound state experiencing two signal sets (cf. (**d**)) are shown in blue on the apo open structure. (**f**) ZZ exchange analysis[1] for exchange (shown in blue) and diagonal signal (shown in red) for Leu6 of Y171W in the Ap5a-bound state yields to k_f = 27 s^{-1} and k_r = 24 s^{-1}. (**g**) ZZ exchange analysis[1] for exchange (shown in blue) and diagonal signal (shown in red) for Gln18 of Y171W in the Ap5a-bound state yields to k_f = 29 s^{-1} and k_r = 25 s^{-1}. (**h**) ZZ exchange analysis[1] for exchange (shown in blue) and diagonal signal (shown in red) for Leu83 of Y171W in the Ap5a-bound state yields to k_f = 25 s^{-1} and k_r = 20 s^{-1}. (**i**) ZZ exchange analysis[1] for exchange (shown in blue) and diagonal signal (shown in red) for Thr89 of Y171W in the Ap5a-bound state yields to k_f = 33 s^{-1} and k_r = 23 s^{-1}. (**j**) ZZ exchange analysis[1] for exchange (shown in blue) and diagonal signal (shown in red) for Gln173 of Y171W in the Ap5a-bound state yields to k_f = 17 s^{-1} and k_r = 5 s^{-1}. (**k**) Kinetic time traces for quenching of the Y171W fluorescence emission caused by Ap5a interaction, recorded at indicated temperatures. (**l**) Quenching of fluorescence emission of Y171W is independent of the Ap5a to Y171W concentration ratio. Data were acquired at 8 °C.

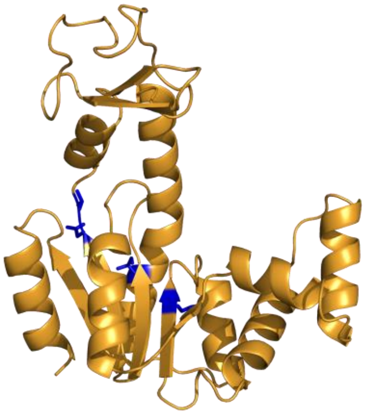

Supplementary Figure 3 Solvent protection for Ap5a saturated Y171W. The residues colored in blue (Leu83, Asp110, Val111 and Ile179) experience significant differences in solvent protection between both directly observed Ap5a-bound conformational states (see **Supplementary Table 2**).

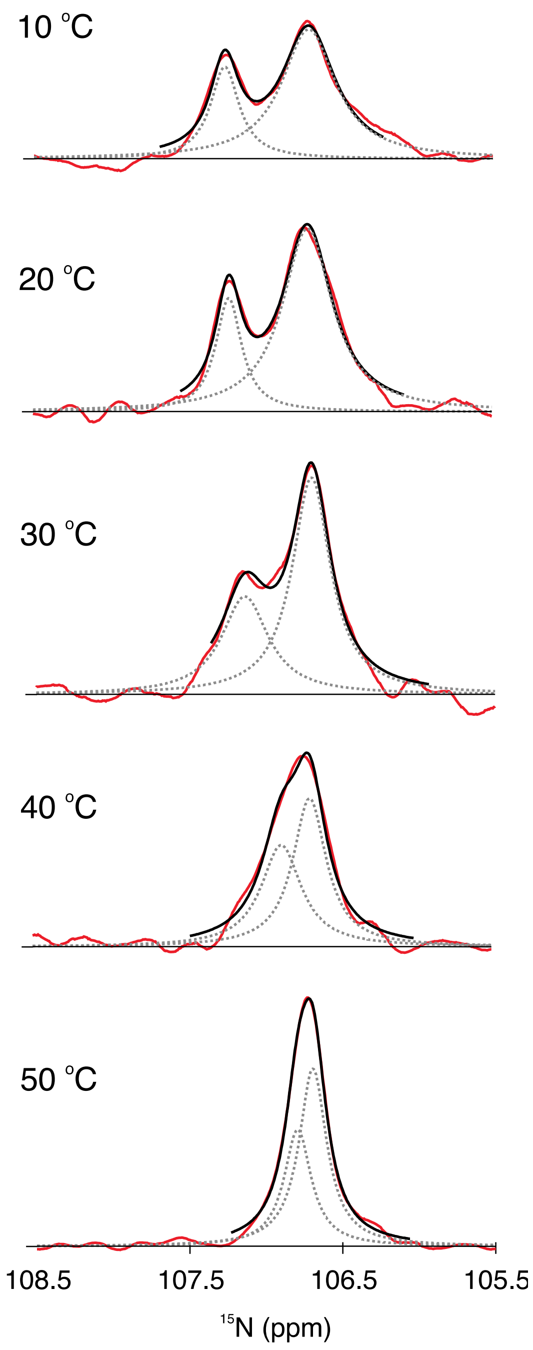

Supplementary Figure 4 Line shape analysis of Thr89 performed for Y171W bound to Ap5a. One-dimensional ^{15}N slices (in red) were extracted from ^{1}H-^{15}N HSQC spectra. Subsequently, a two component Lorentzian function (each in dotted gray) was fitted to individual one-dimensional ^{15}N spectra. Applying McConnell equations possessing the rate of conformational exchange, k_{ex}, results in the spectrum colored in black.

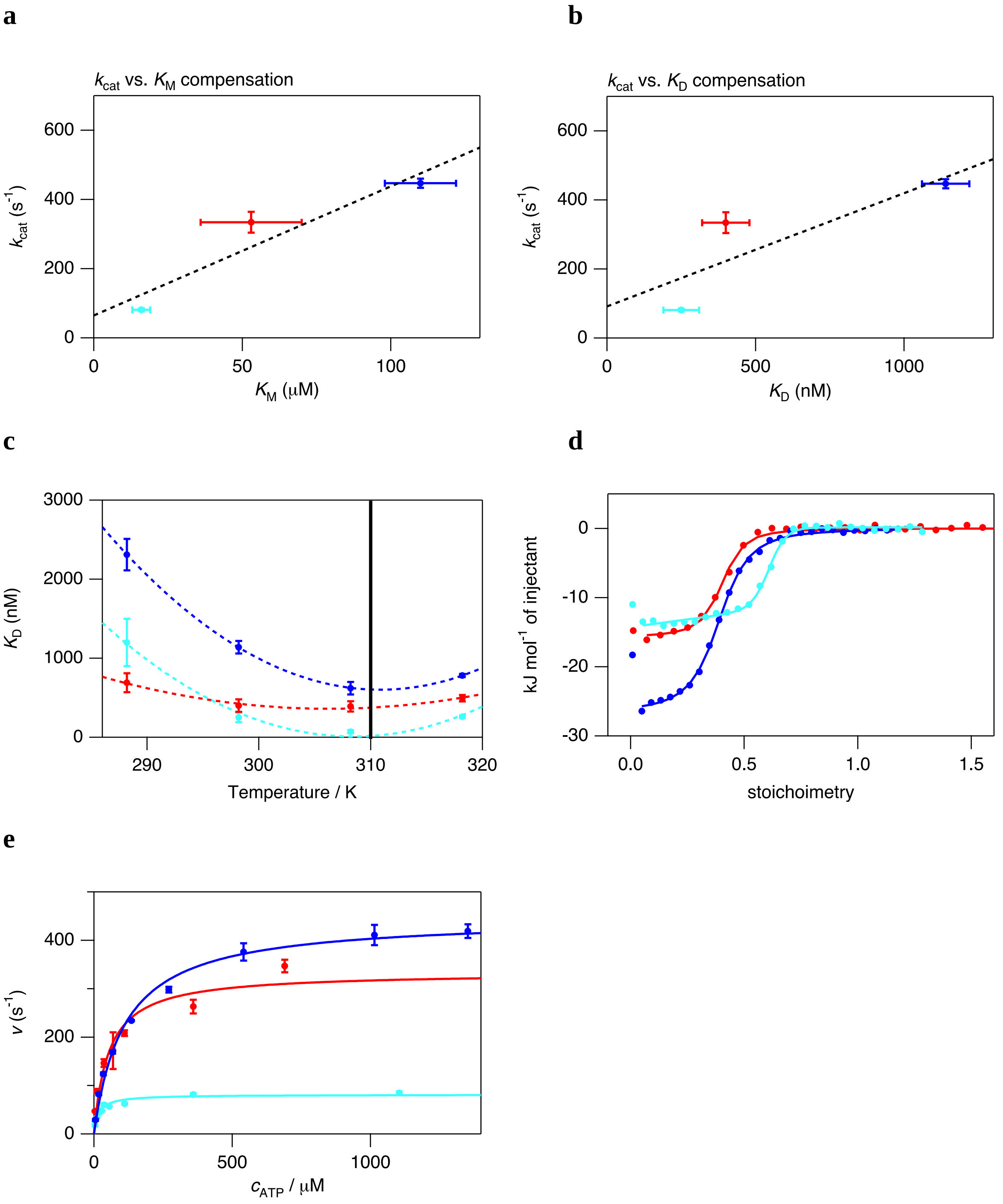

Supplementary Figure 5 Activity and binding characteristics for AdK mutants. (**a**) Catalytic turnover of AdK in direction of ADP formation depends linearly on the Michaelis-Menten constant for ATP binding probed via an enzyme activity assay. The dotted line represents data regression to a linear function. The color code is used for A-E in the following manner: wild type in red, P177A in blue and Y171W in cyan. (**b**) Catalytic turnover of AdK in direction of ADP formation probed via an enzyme activity assay depends linearly on the Ap5a probed via isothermal titration calorimetry. The dotted line represents a linear fit to the data. (**c**) The lowest K_D values, corresponding to the maximum Ap5a binding affinity to AdK is located at

37 °C and is indicated by a vertical line. A polynomial function of third order (dotted lines) was used as a fit to the individual affinity data. Data was acquired by isothermal titration calorimetry. (**d**) Representative isothermal titration profiles for Ap5a binding to AdK recorded at 25 °C. Following standard treatment of ITC data the first titration point in the ITC curve is not considered for the curve fitting procedure. (**e**) Typical profiles for AdK catalyzed ADP formation. Data were acquired at 25 °C.

Supplementary Table 1 Molecular dimension of AdK. The radius of gyration, r_G, was calculated with PyMol and decreases when Ap5a is bound to the enzyme. The diffusion coefficient was determined by NMR spectroscopy (D^{apo}, D^{bound}) and changes for wild type as well as for P177A upon binding to Ap5a according to the change of r_G. The solution property of Y171W bound to Ap5a differs significantly with respect to the corresponding crystallized state.

**pdb code**	**molecule**	r_G **[Å]**	$(r_G^{apo} - r_G^{bound})/r_G^{bound}$ **[%]**	D^{bound}/D^{apo} **[%]**
4AKE	wild type	19.6		
1AKE	wild type + Ap5a	16.9	16	12
4X8H	P177A	19.6		
4X8L	P177A + Ap5a	16.6	18	15
4X8M	Y171W	19.6		
4X8O	Y171W + Ap5a	16.3	20	6

Supplementary Table 2 Solvent accessibility for Ap5a-bound Y171W as determined by MEXICO. Apparent exchange rates, k_{ex}, were calculated according to[2]. Residues showing two chemical shifts at this conditions are indicated in bold face.

aa	k_{ex} [s^{-1}]	Δk_{ex} [s^{-1}]	aa	k_{ex} [s^{-1}]	Δk_{ex} [s^{-1}]	aa	k_{ex} [s^{-1}]	Δk_{ex} [s^{-1}]
**2**	**7.9**	**1.7**	**61**	**19.0**	**22.0**	129	5.2	7.3
**2**	**3.2**	**4.6**	**61**	**35.7**	**23.4**	130	0.2	0.1
5	0.7	0.1	62	3.6	3.0	131	2.4	2.9
**7**	**10.2**	**3.5**	74	4.1	4.7	**135**	**0.9**	**0.1**
**7**	**11.1**	**6.7**	75	32.3	0.8	**135**	**1.7**	**0.1**
8	8.1	6.8	76	4.2	1.4	136	0.7	0.1
11	8.4	4.4	77	4.3	7.3	**137**	**27.7**	**5.9**
12	2.1	0.1	78	0.9	0.1	**137**	**15.4**	**8.7**
12	1.7	0.1	79	15.9	0.8	**138**	**3.7**	**1.8**
16	2.2	0.1	80	2.4	2.4	**138**	**1.1**	**10.8**
19	1.0	0.1	81	4.3	3.3	141	11.5	4.5
20	0.2	0.1	82	1.4	0.1	142	10.5	2.1
23	0.7	0.1	84	0.9	0.1	144	11.9	1.5
25	10.9	0.3	85	16.0	2.5	145	0.5	0.1
26	13.4	13.1	94	13.5	8.7	147	1.5	0.1
28	0.3	0.1	95	0.7	0.1	148	1.7	0.1
29	24.3	22.8	96	1.4	6.0	149	1.7	0.1
30	1.5	0.1	98	0.6	0.1	150	21.5	39.5
31	6.9	2.4	99	7.2	4.0	151	1.9	0.1
**32**	**8.6**	**3.4**	100	1.9	0.1	152	1.0	0.1
**32**	**12.5**	**2.7**	101	5.4	2.0	153	1.7	0.1
33	1.2	0.1	102	8.6	0.9	154	12.1	2.1
34	1.0	0.1	103	17.3	4.1	**155**	**18.1**	**2.9**
35	1.8	0.1	108	8.8	3.2	**155**	**16.0**	**3.6**
38	2.3	1.9	**109**	**2.1**	**8.3**	156	9.0	3.5
39	8.4	1.9	**109**	**1.1**	**0.1**	158	6.1	0.9
41	7.7	1.3	**110**	**1.6**	**0.1**	**160**	**4.1**	**3.2**
42	6.3	1.8	**110**	**13.4**	**9.8**	161	21.7	2.1
43	6.1	1.2	**111**	**3.0**	**10.7**	162	4.4	4.6
44	28.1	2.2	**111**	**42.9**	**31.2**	**163**	**24.2**	**23.0**
45	17.7	0.9	113	3.0	4.3	**163**	**17.8**	**4.7**
46	7.4	2.3	**114**	**7.9**	**10.7**	**164**	**2.4**	**5.4**
47	0.5	0.1	**114**	**3.8**	**9.9**	**164**	**0.8**	**0.1**
49	7.6	2.0	119	0.8	0.1	**167**	**1.0**	**0.1**
**51**	**22.0**	**10.1**	122	0.6	0.1	**167**	**0.9**	**0.1**
**51**	**21.4**	**16.1**	**123**	**6.2**	**3.5**	168	0.2	0.1
52	0.2	0.1	**123**	**2.1**	**2.9**	170	16.2	10.5
53	3.2	4.0	124	6.2	2.8	**171**	**7.4**	**2.9**
56	1.8	1.6	125	3.8	1.7	**171**	**5.0**	**1.8**
59	26.0	3.9	126	33.0	19.1	172	2.7	6.8

aa	k_{ex} [s^{-1}]	Δk_{ex} [s^{-1}]	aa	k_{ex} [s^{-1}]	Δk_{ex} [s^{-1}]	aa	k_{ex} [s^{-1}]	Δk_{ex} [s^{-1}]
174	5.6	1.2	195	8.1	3.8	204	3.8	4.0
175	0.6	0.1	**196**	**10.5**	**8.1**	205	0.6	0.1
**179**	**0.4**	**0.1**	**196**	**14.4**	**11.5**	206	4.5	1.5
**179**	**6.9**	**2.3**	197	1.5	0.1	208	0.9	0.1
185	9.5	13.9	198	1.7	0.1	209	0.6	0.1
190	9.0	3.5	199	13.2	0.6	202	3.4	3.9
191	1.6	0.1	**200**	**3.1**	**3.1**	203	30.6	19.9
192	2.4	4.1	**200**	**5.7**	**8.3**	204	3.8	4.0
193	15.9	5.9	202	3.4	3.9	208	0.9	0.1
194	2.6	10.7	203	30.6	19.9	209	0.6	0.1

Supplementary References

1. Farrow, N.A., Zhang, O., Forman-Kay, J.D. & Kay, L.E. A heteronuclear correlation experiment for simultaneous determination of 15N longitudinal decay and chemical exchange rates of systems in slow equilibrium. *Journal of Biomolecular NMR* **4**, 727-734 (1994).

2. Hofmann, H. et al. Fast Amide Proton Exchange Reveals Close Relation between Native-State Dynamics and Unfolding Kinetics. Journal of the American Chemical Society 131, 140-146 (2008).
